# Systems pharmacology analysis of synergy of TCM: an example using saffron formula

**DOI:** 10.1038/s41598-017-18764-2

**Published:** 2018-01-10

**Authors:** Jianling Liu, Jingjing Liu, Fengxia Shen, Zonghui Qin, Meng Jiang, Jinglin Zhu, Zhenzhong Wang, Jun Zhou, Yingxue Fu, Xuetong Chen, Chao Huang, Wei Xiao, Chunli Zheng, Yonghua Wang

**Affiliations:** 1 Key Laboratory of Resource Biology and Biotechnology in Western China, Ministry of Education, School of Life Sciences, Northwest University, Xi’an, China; 2 Lab of Systems Pharmacology, Center of Bioinformatics, College of Life Science, Northwest A&F University, Yangling, China; 3 State Key Laboratory of New-tech for Chinese Medicine Pharmaceutical Process, Jiangsu Kanion Parmaceutical Co. Ltd., Lianyungang, China

## Abstract

Traditional Chinese medicine (TCM) follows the principle of formulae, in which the pharmacological activity of a single herb can be enhanced or potentiated by addition of other herbs. Nevertheless, the involved synergy mechanisms in formulae remain unknown. Here, a systems-based method is proposed and applied to three representative Chinese medicines in compound saffron formula (CSF): two animal spices (*Moschus*, *Beaver Castoreum*), and one herb *Crocus sativus* which exert synergistic effects for cardiovascular diseases (CVDs). From the formula, 42 ingredients and 66 corresponding targets are acquired based on the ADME evaluation and target fishing model. The network relationships between the compounds and targets are assembled with CVDs pathways to elucidate the synergistic therapeutic effects between the spices and the herbs. The results show that different compounds of the three medicines show similar curative activity in CVDs. Additionally, the active compounds from them shared CVDs-relevant targets (multiple compounds-one target), or functional diversity targets but with clinical relevance (multiple compounds-multiple targets-one disease). Moreover, the targets of them are largely enriched in the same CVDs pathways (multiple targets-one pathway). These results elucidate why animal spices and herbs can have pharmacologically synergistic effects on CVDs, which provides a new way for drug discovery.

## Introduction

TCM has developed over thousands of years and adhered to the holistic therapeutic philosophy^1^. TCM has its own traditional theory of treatment of diseases with formulae containing several herbs, seldom with one single herb, in which the pharmacological functions of a single herb is either prolonged or enhanced, and its side effects decreased by the action of others^2^. This thereby leads to a more pleasant effect for TCM formula than for the herb used alone^3^, which indicates that therapeutic efficacy of formula may profit from synergistic functions of herbal ingredients^2,4^.

Traditional medicine thinks that the synergistic integration may be reflected in the interactions between local and whole body, the body and nature. For modern medicine, herbal synergism has been frequently reported, the synergistic mechanism may result from the enhancement of pharmacokinetics and the potentiation pharmacodynamics. For example, combined administration of drugs which compete for albumin binding will enhance the free drug concentrations, and thus potentiate their actions^2^. However, the mechanism of action of such multicomponent synergy among the interactive molecules, targets, pathways, and even the given diseases remains largely unknown.

In the current work, to explore the underlying synergistic mechanisms from molecule, target and pathway level, a suitable example was taken by animal spices *Moschus* (M) and *Beaver Castoreum* (B) and herb *Crocus sativus* (C) (referred to as MBC) from CSF. CSF is comprised of 13 Chinese medicines, the potential multi-compound pharmacological efficacy on CVDs of it has been validated in previous research^5^. However, the synergy between animal spices and *Crocus sativus* remains ambiguous. Here, this mechanism will be elaborated by MBC. *Moschus* and *Beaver Castoreum* not only serve as the crucial spices but also own astonishing drug potency and high efficacy in combating CVDs, and the curative efficacy of them have been validated in previous research. For instance, with *Moschus* as its main compound, Shexiang Baoxin Pill (SXBXP) has been widely used to treat coronary heart disease, angina pectoris, myocardial infarction, etc.^6,7^, and its therapeutic effect depends largely on *Moschus*. *Beaver Castoreum* was described in the 1911 British Pharmaceutical Codex for raising blood pressure and increasing cardiac output, moreover, *Beaver Castoreum* is also used as an analgesic, anti-inflammatory, and antipyretic, and the activity of it has been considered to be similar with aspirin^8^. Additionally, modern medicine has discovered *Crocus sativus* has the properties of cancer-suppressing, anti-mutagenic, immune-modulating and antioxidant-like and showed positive effects on CVDs^9^.

**Figure 1:**
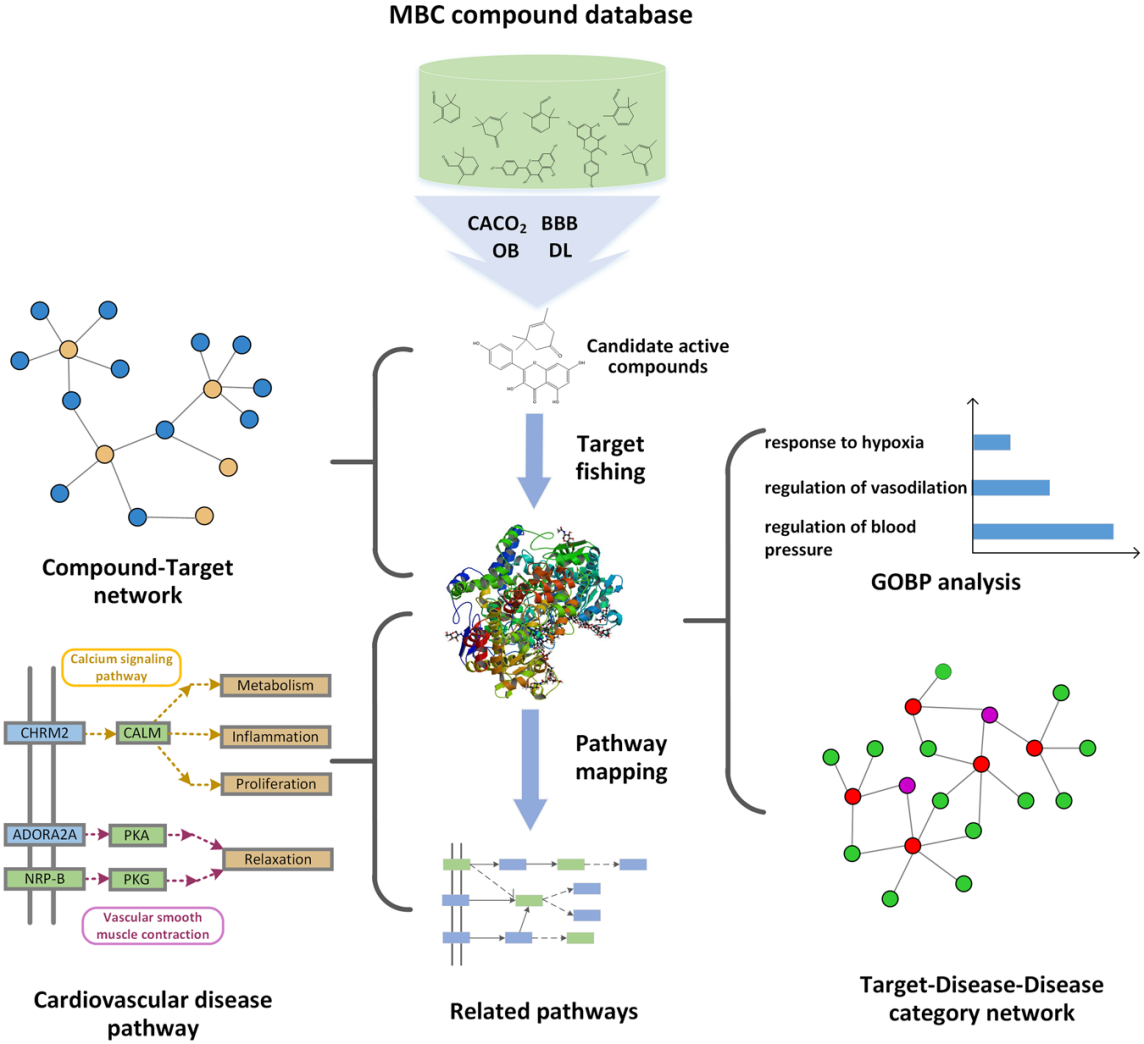
The detailed process introduction of the systems pharmacology research method.

Systems approaches have long been used in pharmacology to understand drug action at the organ and organismal levels^10^. In this study, we have successfully built an integrative systems pharmacology approach and applied it into the large-scale analysis of MBC to dissect the synergy between animal spices and herbal medicine act on CVDs. Briefly, as shown in Fig. 1, firstly, based on ADME evaluation system, we screen out the effective constituents with satisfying pharmacokinetics activity; secondly, we use the active ingredients as the baits to capture their corresponding targets; thirdly, the obtained candidate targets are mapped onto relevant databases to find out the best candidate targets corresponding to CVDs; finally, network construction and compressed ‘CVDs pathway’ is performed to elucidate the synergistic relationship of MBC on CVDs. All these demonstrate that systems pharmacology approach will provide a novel way for dissecting the synergy of TCM and developing new drugs.

## Results

### Uncovering the synergy from multicomponent in MBC on CVDs

As can be seen from Table S1, based on ADME systems, a total of 8, 6 and 28 active constituents are extracted from *Moschus*, *Beaver Castoreum* and *Crocus sativus*, respectively. TCM formula typically incorporates several TCM which contain multi-ingredient may exert a more satisfying effect than a single constituent^2^. CVD is a complex trait disease in which various mediators facilitate overall CVDs pathogenesis by different mechanisms. Therefore, such multi-component therapeutics is regarded as an efficient and rational pattern of therapy devised to combat CVDs^11^. In other words, such multicomponent therapy will elevate the single drug concentrations and thus potentiate their actions, which results in multicomponent synergy. In this section, our method is applied to compare the individual biological function of different ingredients in MBC in treating CVDs.

#### Moschus

Based on ADME properties, 8 active ingredients in *Moschus* are filtered out for further research. Most of the herbs possess many ingredients, but the specific ingredients serve as the pleiotropic active ingredients in generating efficacy in curing CVDs. For example, as the vital constituent of *Moschus*, muscone plays a leading role in repairing ischemia-reperfusion injury in cardiomyocytes due to its oxidation resistance and anti-apoptosis effects^12^. Besides, muscone exhibits beneficial effects on improving cardiac function and attenuating myocardial remodeling after myocardial infarction^13^.

#### Beaver Castoreum

Based on ADME properties, 6 active compounds in *Beaver Castoreum* are screened out. *Beaver Castoreum* has been used for medicines and perfumes since time immemorial^14^. The ingredients of *Beaver Castoreum* contain volatile oil, castorin, salicin and ethylphenol, which are suggested to acting on neuropathy to reduce the symptoms of pain and spasmodic, as is described that *Beaver Castoreum* is used for raising blood pressure and increasing cardiac output^8^. As the important ingredient, salicin as a precursor of acetylsalicylic acid after hydrolyzed, acetylsalicylic acid constitutes the most frequently used antiplatelet drug, it has been used for prevention of CVD in patients with diabetes, ischemic stroke^15^, besides, acetylsalicylic acid is regarded as a possible preventive factor of coronary thrombosis^16^.

#### Crocus sativus

28 potential compounds in this herb are filtered out for further research. The biological functions of the specific ingredients in the herb exert complementary roles with other two animal spices in treating CVDs. For instance, the pleiotropic health-promoting effects of crocin have been reported extensively in pre-clinical investigation, such as anti-oxidation^17,18^, antidepressant^19^, antitumor^20–22^ and protective effects on heart and nerves^23–27^. Previous studies have demonstrated that crocin could be used for the treatment of CVDs due to its potential antihyperlipidemic effects^28^, inhibition of calcium channels^29^ and obviously antioxidant reactivity^30^. Advanced pharmacological studies also have verified its brain protection effect in cerebral ischemia^31^ and antitumor effects^32^. Recently, it has been reported that another ingredient crocin1 plays a major role in the regulation of plasma lipid by selectively inhibiting pancreatic enzyme activity due to poor oral bioavailability of it^33^. Crocetin serves as the pleiotropic active ingredients in *Crocus sativus*, and have been confirmed to exhibit effective neuroprotective activity^34^, antioxidant^35^, anti-inflammatory^36^, anti-atherosclerotic^37^, improving insulin resistance^38^, which probably play significant role in curing CVDs.

Thus, the specific constituents in MBC serve as the potential compounds in CVDs related symptoms therapy. In summary, the different biological function of different ingredients in MBC suggesting that the three TCMs with complementary pharmacological roles in treating CVDs. It is reasonable to speculate that the mutual enhancement of MBC could produce a complementary synergistic effect, which could be achieved through different modes of action.

### The targets fishing and identification

**Figure 2:**
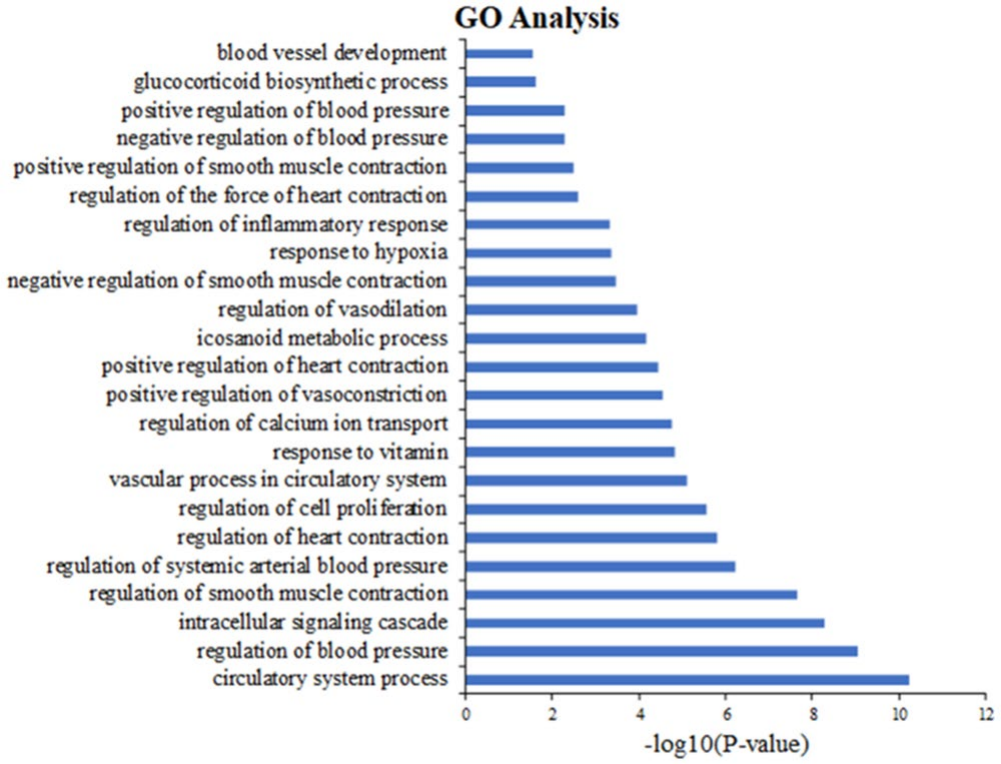
Gene Ontology (GO) analysis of potential targets. The y-axis shows significantly enriched ‘Biological Process’ (BP) categories in GO associated with the targets, the x-axis shows the enrichment scores of these terms (P Value ≤ 0.05).

For TCM, pharmacological effects of one constituent are usually enhanced by other one or more in a formula. The nature of such synergistic effect is probably result from the bioactive ingredients acting on either the same receptor target or a similar physiological system^2^. With the aid of the algorithm, we have captured the potential targets: 33, 25, and 46 of related compounds which from *Moschus*, *Beaver Castoreum* and *Crocus sativus*, respectively (as shown in Tables S2 and S3). As shown in Fig. 2: In the succedent GOBP enrichment analysis, we find out that various targets are strongly associated with various biological processes of the occurrence of CVDs, such as vasodilation regulation, smooth muscle contraction regulation, blood pressure regulation and positive vasoconstriction regulation. These biological processes are all closely associated with the pathogenesis of CVDs. Manifesting that the compounds in MBC can act on functional diversity targets, and thus improve polypharmacology in combating CVD.

### Revealing the pharmacology synergy from network level

**Figure 3:**
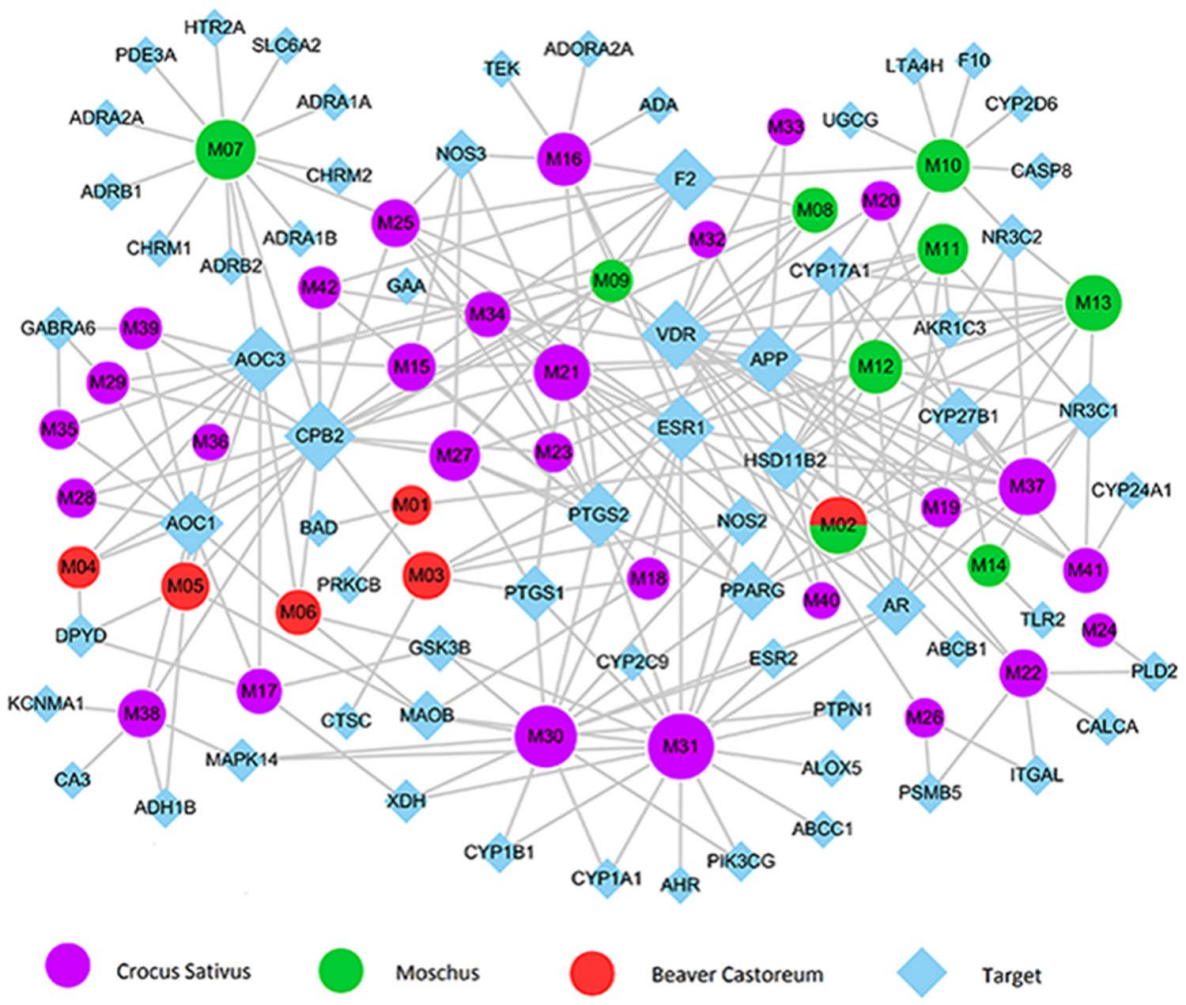
Compound-target network. Blue nodes represent potential drug targets, 670 purple, green and red nodes remark drug ingredients of Crocus sativus (purple), 671 Moschus (green) and Beaver castoreum (red), respectively. And each edge represents the interaction between them. Node size is proportional to its degree.

In order to decode the synergic mechanism of MBC in combating CVDs, all CVDs-related targets are used to establish the compound-target (C-T) network, as shown in Fig. 3, which consists of 108 nodes (42 compounds associated with 66 potential target proteins) and 242 edges, the degree value of active compounds and potential targets was represented by the node size. As a result, an average degree of 5.8 for compounds and 3.7 for targets, respectively, which elaborate the polypharmacology characteristic of the multicomponent in MBC. The polypharmacology synergy may be reflected in the specific targets of the different compounds have different pharmacological roles in treating CVDs, and the common targets can simultaneously act on specific biological process of CVDs. Thus, a complementary synergistic effect may be exerted from the specific and common target of the compounds in MBC.

#### Specific Targets of the Compounds in MBC. Moschus

For this animal spice, 8 candidate compounds are probably related to 33 potential targets, of which 15 targets are validated own close relationship with CVDs, such as VDR (Vitamin D3 receptor) and CPB2 (Carboxypeptidase B2), all the targets have been validated with amazing therapeutic effect for cardiovascular complications. F2 (Prothrombin) is reported to be closely associated with stroke, venous thrombosis and myocardial infarction in a large group of American men^39^. HTR2A (5-Hydroxytryptamine 2 A Receptor) antagonists enable to inhibit vasoconstriction and thrombosis^40^.

#### Beaver Castoreum

For this animal spice, a total of 25 targets are captured corresponding to 6 potential compounds, this means that one compounds hits 4.1 targets on average, which indicates the polypharmacology characteristic of TCM. Among 25 targets, 12 of them are validated with therapeutic function in CVDs. For example, VDR is proven to be with the function of regulating vasodilatation; ADRB1 (Beta-1 adrenergic receptor) and ADRB2 (Beta-2 adrenergic receptor) serve as the vital role in the pathology and biology process of arrhythmia.

#### Crocus sativus

There are 46 potential targets are obtained from 28 bioactive compounds in this herb, this means that a constituent targets 1.6 target proteins on average, which manifests the polypharmacology feature of TCM., 14 targets among 46 has already been proved with pharmacological activity on CVDs. For instance, PTPN1 (Tyrosine-protein phosphatase non-receptor type 1), PTGS2 (Prostaglandin G/H synthase 2) are considered to be a potential therapeutic target in the formation of thrombosis, the active compounds can inhibit blood clotting by controlling these proteins, activate fibrin dissolution, inhibit platelet aggregation, reduce thrombus viscosity and finally achieve the purpose of inhibiting thrombosis^40–43^. It suggested that the CALCA (Calcitonin gene-related peptide 1) with potential effect in blood pressure regulation *in vivo* ^44^.

#### Shared targets of the compounds in MBC

There exist more than 20 targets simultaneously shared by more than one compounds in MBC. For example, the common target PTGS2, which is related to heart failure^45^, can be modulated by 2 compounds in *Beaver Castoreum*, 2 in *Moschus* and 8 in *Crocus sativus*, respectively. Another important target AOC3 (Membrane primary amine oxidase) with amazing therapeutic effect for cardiovascular complications is targeted by 3 ingredients in *Beaver Castoreum*, 4 in *Moschus* and 6 in *Crocus sativus*, respectively. Similarly, HSD11B2 is impacted by 2 constituents in *Beaver Castoreum*, 4 in *Moschus* and 2 in *Crocus sativus*, respectively. These suggest that several constituents in MBC can simultaneously hit to the same targets in a given formula, thus lead to synergy effect on CVDs. In addition, ESR1 (Estrogen receptor) targeted by 13 active compounds in MBC, PPARG (Peroxisome proliferator-activated receptor gamma) targeted by 8 potential compounds in MBC. Similarly, this suggests that synergistic or complementary effects of the animal spices and *Crocus sativus* in the treatment of CVDs.

#### Shared targets act on CVDs

**Figure 4:**
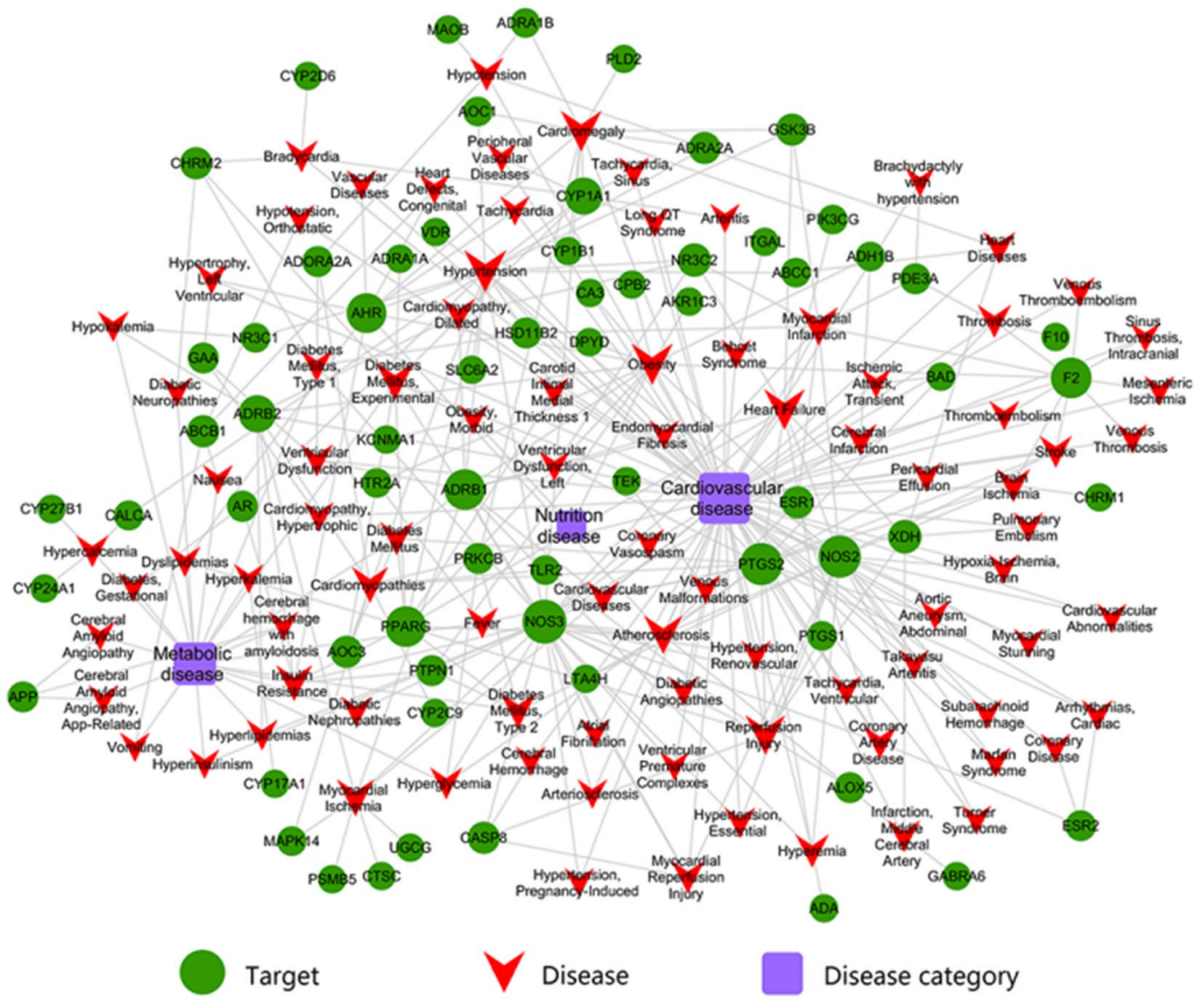
Target-disease-disease category network. Green nodes represent potential drug targets, red nodes remark the CVDs-related diseases, blue nodes represent disease category, each edge represents the interaction between them. Node size is proportional to its degree.

To further interpret the synergistic action mechanism of biologically active targets, the interaction network of related CVDs and their candidate targets are taken into a target-disease-disease category (T-D-Dc) network, which assembled by 155 nodes (66 targets, 89 relevant CVDs) and 331 edges. The occurrence of CVDs featured as multi-factor interaction and all kinds of diseases influence each other, as the Fig. 4 shows: the whole diseases are divided into three categories (cardiovascular diseases, metabolic diseases and nutritional disease) due to their direct or indirect mode of action. These potential shared targets are involved in the different stages of CVDs, for instance, PTGS2 plays a significant role in the treatment of heart failure, a previous research showed that PTGS2 is one of the threatening moderator in the CVDs morbidity, Prostaglandin E2 as a product involved in the processes of inflammation in vascular smooth muscle cells^45^. Shared NR3C2 (Mineralocorticoid receptor) was proved being an regulatory factor in the regulation of blood pressure due to its function of promoting the salt retention in kidney^46^. Especially for HSD11B2, shared by the three drugs was validated to be a participant in several biological processes such as lipid biosynthetic process, the regulation of systemic arterial blood pressure, oxidation-reduction reaction and hormone synthesis and metabolism and so on, as previous study shows that the inhibition activity of HSD11B2 will stimulate mineralocorticoid receptor and finally elevate blood pressure^47^. As is showed that AOC3 is an active factor in adipocytes and smooth muscle cells, it has been proven to be an anti-inflammatory target in the drug development process^48^. Altogether, the candidate targets play a curative effect by regulating different biological processes in treating CVDs.

### Uncovering the synergy from pathway level

**Figure 5:**
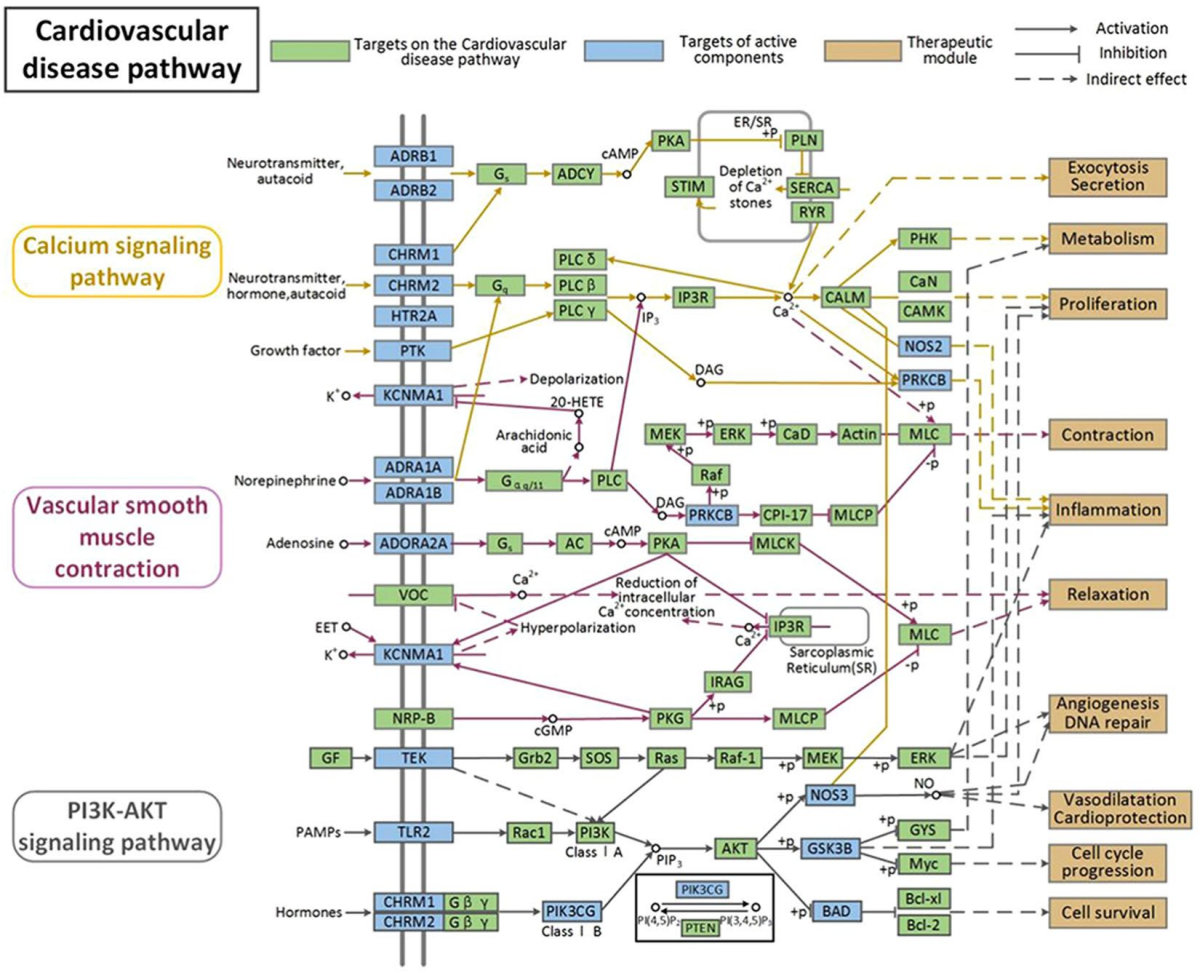
CVDs pathway. Distribution of protein targets of herbs on the compressed ‘CVDs pathway’. Three pathways (colorless rectangle) form the compressed CVDs pathway. Light green rectangles remark targets on the CVDs pathway. Light blue rectangles represent targets of active compounds. Brown rectangles remark 683 therapeutic module. Arrows indicate activation, T-arrows indicate inhibition.

To further probe into the synergistic mechanism of MBC in treating CVDs from pathway level, a ‘CVDs pathway’ based on the existing knowledge of CVDs pathology is assembled in this section. Those target proteins exhibit conspicuously functional correlation closeness to the targets related to CVDs pathway. As shown in Fig. 5, this CVDs pathway can be broken up into several CVDs-related functional modules such as angiogenesis, inflammation, contraction, metabolism, DNA repair and so on. One or more target of single signaling pathway may influence on another pathways synchronously, as a result, the cross regulation of multiple pathways is generated^49^. What’s more, individual ingredient of MBC could act on distinct targets of the same pathway, which indicate that the biological process is implemented by the various actions of different targets. The common targets from MBC could act on the CVD-related functional modules, the interaction between them is achieved through the regulation of the common target of these two pathways, thereby producing synergy effect in fighting CVDs. To dissect the potential synergistic mechanisms associated with the disease pathway, several representative functional modules are extracted and the analysis results are as follows.

#### The different targets act on myocardial contraction module

As shown in Fig. 5: herbal constituents may improve myocardial contractility by disturbing calcium signaling pathway. For example, M08 (in *Moschus*) act on HTR2A, HTR2A as a basic activator of CALM. CALM as a multifunctional receptor protein plays an important role in the intracellular calcium signaling pathway. CALM can regulate myocardial contraction function by relying on the intracellular Ca^2+^ concentration^50^. Besides, studies have shown that in the case of specific pathophysiology, HTR2A antagonists can inhibit vasoconstriction and thrombosis^40^.

As a leading risk factor of CVDs, myocardial contraction is related to several proteins in the CVDs-pathway. PRKCB (Protein kinase C beta type) was predicted to be suppressed by M01, as previous experimental evidence demonstrated that PRKCB as a potential regulator of Ca^2+^ handling and cardiomyocyte contractility, and pharmacological inhibition of PRKCB, may serve as a novel therapeutic strategy for enhancing cardiac contractility in certain stages of heart failure^51^.

#### The Shared targets act on angiogenesis module

Angiogenesis as a vital and efficient therapeutic protocol in combating relevant CVDs, which overcome the difficulty of neovascular insufficiency leads to insufficiency of heart supply. This therapeutic module not only effectively promotes the restoration of damaged tissue, but also improves tissue ischemia, as shown in Fig. 5: some shared proteins involve in sustained angiogenesis are regulated by acting on the PI3K-Akt signaling pathway to generate the remedial effect of neovascular insufficiency. We can see, as the vital factor of angiogenesis, M16 in *Crocus sativus* acted on TEK (Angiopoietin-1 receptor) was observed to increase NOS3 (targeted by M11 in *Crocus sativus* and M04 in *Beaver castoreum* and M16 in *Moschus*) and GSK3β (targeted by M30 in *Crocus sativus* and M07 in *Moschus*) level, respectively. Previous studies have shown that the increase of GSK3β suppresses the process of angiogenesis, however, down regulation of GSK3β facilitates the shaping of capillaries^52^. Besides, as an angiogenesis regulator, NOS3 is capable of producing NO and has the ability to regulate angiogenesis *in vivo* and *in vitro* ^53^. Usually, angiogenesis is a complex process, the potential protein targets act on signaling pathway to achieve the efficient therapeutic effects.

#### The cross regulation of MBC on vasodilatation and cardioprotection module

The synergistic mechanism of spice and herb may be reflected in the regulating intracellular signaling cascades through the management of different proteins from MBC. As can be seen in Fig. 5, CHRM1, CHRM2 (M8 in *Moschus*) as the major regulators of PIK3CG (M30 in *Crocus sativus*), the up-regulation PIK3CG will promote the occurance of Akt-mediated NOS3 (M02 in *Beaver castoreum*) phosphorylation. Under physiological conditions, the nitric oxide (NO) in the blood vessels mainly generate in endothelial cells and cardiomyocytes by the endothelial form of nitric oxide synthase (eNOS or NOS3), which is crucial for cardiovascular health^54^, such as promoting vasodilatation, inhibiting platelet aggregation and adhesion as well as involving in the activation of endothelial cells. Additionally, the signaling of eNOS or NO plays a major part in cardioprotection^55^, as a work found out that eNOS phosphorylation is a pivotal molecular switch in vasodilation and cardioprotection^56^. Akt-mediated eNOS phosphorylation was closely associated with the treatment of various cardioprotective traits such as ischemic pre and post-conditioning, with eNOS-derived NO from the endothelium serves as a critical role^57,58^. We speculate the combination of MBC will show great promise to exert synergistic complementary effect in CVD therapy.

#### Cross-talk between pathways

In the compressed CVDs pathway, several signaling pathways interacted with each other by one or more target proteins of one signaling pathway produce effect on another pathway simultaneously^49^. As shown in Fig. 5, there exists two typical cross-talk between PI3K-Akt/vascular smooth muscle contraction signaling pathway and calcium signaling pathway, thanks to the mutual interaction between them which is performed through the regulation of the shared target involved in the two pathways.

For instance, these two signaling pathways in the diagrammatic drawing are shown to band together to regulate intracellular signal cascades through the control of protein PRKCB, PRKCB is recognized as a proximal regulator of cardiac contractility and Ca^2+^ disposing in cardiomyocyte. Activation of PRKCB will act on the inflammation in calcium signaling pathway, and the pharmacological inhibition of PRKCB may serve as a novel therapeutic strategy for enhancing cardiac contractility in the vascular smooth muscle contraction. Thus, it is reasonable to speculate the synergy of MBC main be generated from pathway level.

## Discussion

The prevalence of CVDs and the incapability of existing antagonistic therapy in confronting with the complex features disorder make the powerful treatment strategies controversial. TCM formula through potential active ingredients act on the multi-target, multi-pathway in the biological network to produce synergistic effect, so as to interfere with the occurrence and development of disease, and ultimately achieve the therapeutic effect^11^. In TCM, multiple drugs are often used together to execute therapeutic actions, interestingly, as important constituents, animal spices show synergistic pharmacological activity with other herbs in the formula, such as CSF. In spite of this, the exploration of synergistic action mechanisms of animal spice and herb in TCM remains elusive.

The synergistic effect of multicomponent therapy is usually carried out in a specific case study^59^, which indicates labor-intensive and time-consumption. Additionally, the biological functions are still elusive due to the unknown bioactive compounds, the potential targets and the related pathways of MBC, what the synergistic action modes among them are still ambiguous, which make it extremely difficult to elucidate the synergistic mechanisms by traditional pharmacological methods. Here, a systems pharmacology approach is proposed to insight into the synergistic pharmacological mechanisms of the animal spices *Moschus*, *Beaver castoreum* and the herb *Crocus sativus* (MBC) in treating CVDs.

In this study, on the basis of CVDs’ biological process and TCMSP analysis platform, 42 active compounds are extracted from MBC and 66 corresponding candidate targets are achieved by drug targeting model. To elaborate the credibility of ADME evaluation system and the robustness of WES drug targeting model, then GO enrichment analysis is applied to capture the close relationships between potential targets and CVDs to further confirm the therapeutic mechanism of the herbal ingredients through modulating proteins in the biological process. Finally, C-T network, T-D-Dc and CVDs pathway are assembled to dissect the synergistic mechanisms of MBC in combating CVDs.

In summary, the above results offer insights into the synergistic effect between animal spices and *Crocus sativus*, which could be achieved through the following tactics: (1) the various pharmacological roles of the different ingredients in MBC enable to exert a complementary synergistic effect; (2) multi-compound of MBC acts on the same potential target; (3) multi-compound acts on several different targets in related or even the same CVD-pathway; (4) one or more target of a single signaling pathway influence another one even more pathway simultaneously. All these demonstrate that spices and herbs in TCM show effective synergy activity in regulating CVDs in various pathological relevant biological processes. Therefore, the mutual assistance of different ingredients could be achieved from the distinct mode of actions to perform a complementary pharmacological synergy. The strategy provides a potential way for the rational discovery of new drugs.

## Materials and Methods

### Molecular database construction

All of the chemical ingredients of MBC in the study are derived from our previously established analytical platform: traditional Chinese medicine systems pharmacology database (TCMSP) (http://lsp.nwsuaf.edu.cn/)^60^, we have collected 44, 17 and 53 potential ingredients of *Moschus*, *Beaver Castoreum* and *Crocus sativus*, respectively. In herbal ingredients, glycosides are usually hydrolyzed to release aglycone which is then absorbed in the intestinal mucos^61^, thus, use _qt marked those with no aglycone for further analysis. In this section, based on pharmacokinetic (the absorption, distribution, metabolism, excretion (ADME) properties of compounds) evaluation to extract the chemical ingredients with favorable pharmacokinetics properties^62^. Notably, the ADME evaluation systems have been successfully applied to extract the chemical ingredients with favorable pharmacokinetics properties in Tianshu Formula^63^. This ADME systems including PreOB (predict oral bioavailability), PreDL (predict drug-likeness) and PreCaco-2 (predict Caco-2 permeability). The filtered active compounds must meet the three conditions simultaneously.

#### OB Prediction

OB refers to the ratio of the oral dose after dissolved, absorbed into the body and then reaches the circulation system with their pharmacological activity retained, which is a crucial criterion in filtering the active ingredients with favorable pharmacokinetics properties. In the study, we brought up a robust in-house model OBioavail 1.1^64^ that it integrated the metabolism and transport information is employed to calculate the OB values of all ingredients. And the threshold of OB value is positioned as 30% by the following conditions: first, extracting information from the studied TCM should be as much as possible with the least number of molecules; second, correctly explaining the obtaining model by the reported pharmacological data. Therefore, the compounds with OB ≥ 30% are extracted for further analysis^65^.

#### DL Prediction

In the discovery of new drug, Drug-likeness (DL), a qualitative concept, is usually used. which refers to the expected compounds whether possess the functional groups and/or has physical properties consistent with the majority of known drugs^66^, which helps to optimize pharmacokinetic and pharmaceutical properties, such as solubility and chemical stability^67^. In this study, a self-constructed model (Tanimoto coefficient)^68^ is performed to predict the drug-like characteristics of the expected molecules. The formula of calculating drug-likeness index is defined as follows:

1 $$T(A,B)=\frac{A\cdot B}{{|A|}^{2}+{|B|}^{2}-A\cdot B}$$

where A shows the molecular properties of herbal ingredients, and B shows the average molecular properties of all compounds in DrugBank database (http://www.drugbank.ca/), which based on Dragon soft descriptor. As a necessary molecular descriptor, DL follows the following principle: The higher DL index a herbal ingredient has, the larger possibility it may possess certain biological properties. Finally, the ingredients with suitable DL index (DL ≥ 0.18) are chosen as the candidate compounds for further research.

#### Caco-2 Permeability Prediction

For the sake of screening effective orally administered constituents, oral absorption properties of drugs across the intestinal epithelial cell barrier play a vital role in the process. So in our study, the *in silico* Caco-2 permeability model^69^ is employed to select compounds that possess good permeabilities for further research. Finally, the ingredients with Caco-2 value less than −0.4 are deleted.

So as to obtain a more complete and accurate result, some certain rejected ingredients, which have relatively poor pharmacokinetic properties, but are the most abundant and effective ingredients of certain TCM and with the direct affection of the disease are also selected as the active components for further research.

#### Drug target fishing

As we know, traditional information retrieval approaches of therapeutic targets are complicated and tedious^70^. In our study, drug target fishing is implemented by a large-scale systematic drug targeting tool (SysDT)^71^ and recently developed computational model named weighted ensemble similarity (WES).

For SysDT, on the basis of two mathematical tools, Random Forest (RF) and Support Vector Machine (SVM), the model can ascertain the compound-target interaction profiles comprehensively^71^. These two models exert great suitable efficacy in predicting the drug-target mutual effects, with a concordance of 82.83%, a sensitivity of 81.33%, and a specificity of 93.62%. In this study, the compound target interactions with SVM score ≥ 0.8 and the RF score ≥ 0.7 are selected for further research. Another computational model WES, which integrated CDK parameter, Dragon parameter and CDK-Dragon mixed parameters is clearly proposed to predict drug direct targets^72^. This hybrid parametric model is superior to any single one in predicting the binding (sensitivity 85%, SEN) and non-binding (specificity 71%, SPE) relationships, and the average areas under the receiver operating curves (ROC, AUC) of 85.2% and an average concordance of 77.5%. Notably, both methods have been successfully applied to the targets prediction of Xijiao dihuang decoction for the treatment of viral hemorrhagic fever^73^, Reduning injection in combating inflammation^74^ and Qubaibabuqi formula for vitiligo pathogenesis^75^.

The obtained protein targets are then mapped to the UniProt Database (http://www.uniprot.org/) for normalization. Subsequently submitted to the Pharm Gkb Database (https://www.pharmgkb.org/)^76^, the Therapeutic Targets Database and the Comparative Toxicogenomics Database (http://www.ctdbase.org/)^77^ to remove redundant and erroneous targets to ensure the accuracy of the target database. Finally, to address the functional annotation of targets, we imported the potential targets into the DAVID (http://david.abcc.ncifcrf.gov) for Gene Ontology (GO) enrichment analysis. In this study, GOBP is employed to clarify the interactions between targets and pharmacological effects. The GO terms with *p* value ≤ 0.05 are filtered out for further analysis. Then functional annotation clustering analysis is implemented from the obtained terms to further analyze the pharmacological related biological processes.

### Network construction

To reveal the multiple targets and multiple functions synergistic therapy characteristic of the active constituents in fighting CVDs. We construct compound-target (C-T) network, target-disease-disease category (T-D-Dc) network to systematically and globally reveal the associations between herbs and protein targets in treating CVDs.

In this section, the networks are generated by Cytoscape^78^, which is a popular bioinformatics package for biological network visualization and data integration. In the generated network, active compounds, effective targets and given diseases are represented by nodes, and the interactions between them are represented by edges. Besides, a vital topological parameter-degree is proposed by the plugin Network Analyzer of Cytoscape, since degree is the typical parameter for screening compound targets, we just take degree for network analysis as previous study^79^. The size of nodes represents the degree defined as the number of edges connected to the node.

### Pathway construction

To explore the synergistic biological effect of MBC on CVDs from the pathway level, an incorporated “CVDs pathway” is established based on the current knowledge of CVD pathology. The potential targets contained in signaling pathways are closely related to the biological process of CVDs. The pathway-based combinatorial therapies may become a promising method for the optimization of therapeutic efficacy. The “CVDs pathway” can be obtained from the approach as follows: firstly, the obtained human protein targets related to CVDs are collected to be inputted into the Kyoto Encyclopedia of Genes and Genomes database (KEGG, http://www.kegg.jp/) to acquire the information of signaling pathways. Secondly, an incorporated “CVDs pathway” is assembled by picking out the potential targets with tags which indicate close relationship between ingredients and disease.

### Data availability

The datasets generated during and/or analysed during the current study are available from the corresponding author on reasonable request.

### Equipment and settings

Visio of Microsoft Office2013 was used in Fig. 1.

Excel of Microsoft Office2013 was used in Fig. 2.

Cytoscape 3.2 was used in the Fig. 3.

Cytoscape 3.2 was used in the Fig. 4.

Visio of Microsoft Office2013 was used in the Fig. 5.

## Electronic supplementary material

Supplementary information
