# Supplementary material for: Systems pharmacology analysis of synergy of TCM: an example using saffron formula
Source: Sci Rep. 2018 Jan 10;8:380. doi: 10.1038/s41598-017-18764-2 (PMC5762866; doi:10.1038/s41598-017-18764-2)
Supplement: Supplementary file 1 — Supplementary information [file 41598_2017_18764_MOESM1_ESM.pdf]

Systems pharmacology analysis of synergy of TCM: an example using saffron formula

Jianling Liu[#1], Jingjing Liu[#1], Fengxia Shen[1], Zonghui Qin[1], Meng Jiang[1], Jinglin Zhu[1], Zhenzhong Wang[3], Jun Zhou[3], Yingxue Fu[2], Xuetong Chen[2], Chao Huang[2], Wei Xiao[3*], Chunli Zheng[1*] and Yonghua Wang[1*]

Table S1 Chemical information of 42 active compounds and their network parameters.

NO.	Compound	OB(%)	DL	CACO 2	Degree	Structure	Herbs
M01	Castorin	10.87	0.40	-0.02	3	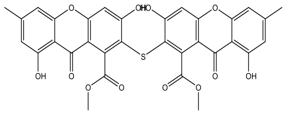	*Beaver castoreum*
M02	Cholesterol	37.87	0.68	1.31	10	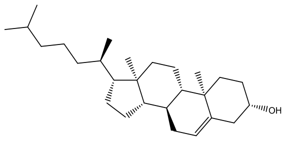	*Beaver castoreum* *Moschus*
M03	Salicin	8.38	0.16	-1.05	6	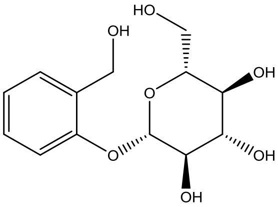	*Beaver castoreum*
M04	Salicin_qt	60.32	0.02	0.89	4	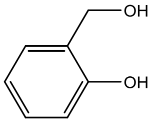	*Beaver castoreum*
M05	Benzoic acid	35.2	0.02	0.85	6	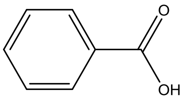	*Beaver castoreum*
M06	Ethylphenol	72.56	0.02	1.60	5	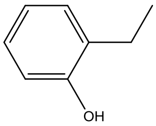	*Beaver castoreum*
M07	Muscopyridine	15.31	0.12	1.76	13	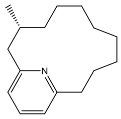	*Moschus*
M08	Muscone	36.63	0.1	1.43	5	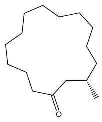	*Moschus*
M09	Normuscone	39.54	0.09	1.39	4	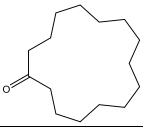	*Moschus*

M10	Muscopyran	63.08	0.64	-1.71	8	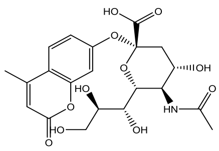	*Moschus*
M11	5a-Androstanedione	14.72	0.35	0.71	7	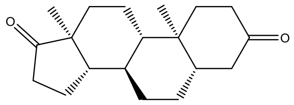	*Moschus*
M12	Cholest-4-ene-3-one	7.45	0.68	1.45	8	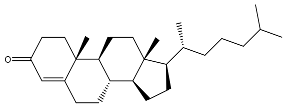	*Moschus*
M13	Dehydroepiandrosterone	33.86	0.35	0.67	9	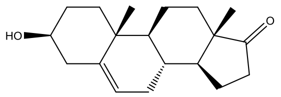	*Moschus*
M14	Cholesteryl ester	45.15	0.39	1.59	4	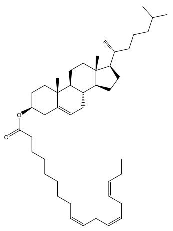	*Moschus*
M15	Crocetin dialdehyde	38.46	0.19	1.16	6	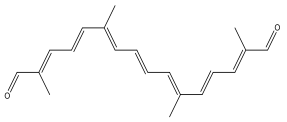	*Crocus sativus*
M16	Adenosine	19.90	0.18	-1.52	8	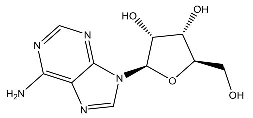	*Crocus sativus*
M17	Adenosine_qt	59.70	0.03	-0.26	5	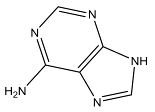	*Crocus sativus*
M18	Anthocyanins	55.28	0.11	1.80	4	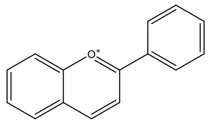	*Crocus sativus*
M19	Carotene-a	36.04	0.58	2.30	3	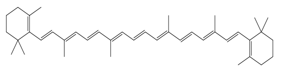	*Crocus sativus*
M20	Carotene	37.71	0.59	2.27	3	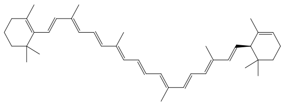	*Crocus sativus*
M21	Crocetin	44.03	0.26	0.56	9	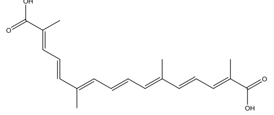	*Crocus sativus*

M22	Crocin	2.48	0.12	-4.40	6	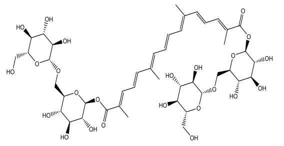	*Crocus sativus*
M23	Crocin_qt	33.44	0.26	0.44	3	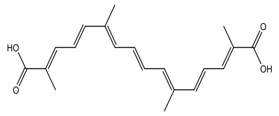	*Crocus sativus*
M24	Crocin1	3.01	0.12	-4.89	1	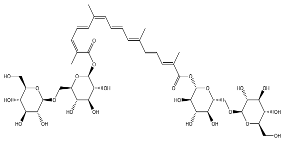	*Crocus sativus*
M25	Crocin1_qt	45.07	0.26	0.46	6	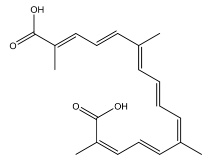	*Crocus sativus*
M26	Crocin2	2.90	0.21	-3.18	3	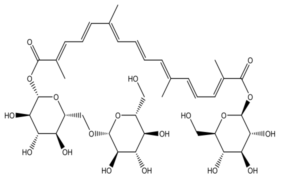	*Crocus sativus*
M27	Crocin2_qt	41.40	0.26	0.46	7	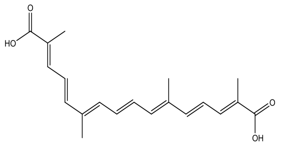	*Crocus sativus*
M28	Foron	32.29	0.02	1.27	3	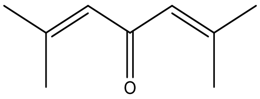	*Crocus sativus*
M29	Isophorone	44.98	0.03	1.28	4	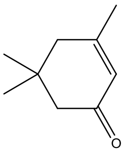	*Crocus sativus*
M30	Isorhamnetin	7.02	0.31	0.12	15	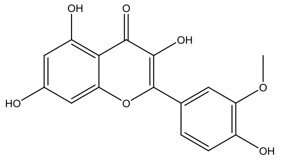	*Crocus sativus*
M31	kaempferol	69.61	0.24	0.15	19	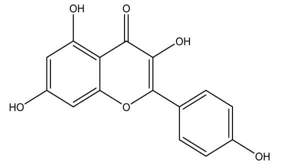	*Crocus sativus*

M32	Lycopene	36.18	0.51	2.41	2	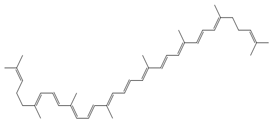	*Crocus sativus*
M33	Phytofluene	43.18	0.50	2.28	2	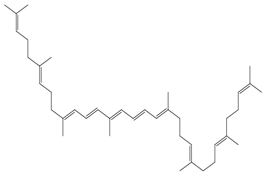	*Crocus sativus*
M34	Picrocrocin	22.42	0.23	-0.82	5	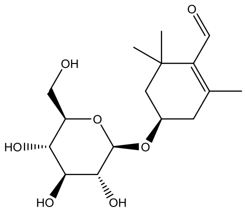	*Crocus sativus*
M35	Picrocrocin_qt	38.42	0.04	0.71	3	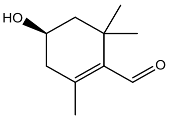	*Crocus sativus*
M36	Safranal	39.81	0.04	1.42	2	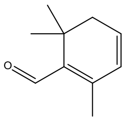	*Crocus sativus*
M37	Stigmasterol	43.83	0.76	1.31	10	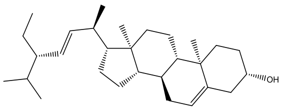	*Crocus sativus*
M38	Catechol	29.35	0.02	1.08	6	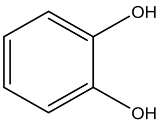	*Crocus sativus*
M39	β-Phorone	46.35	0.03	1.30	4	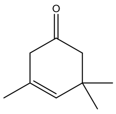	*Crocus sativus*
M40	Tetrahydrolycopene	33.02	0.49	2.41	2	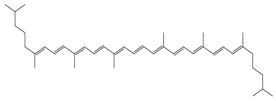	*Crocus sativus*
M41	Zeaxanthin	14.18	0.54	1.04	5	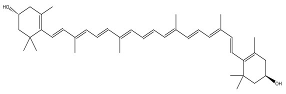	*Crocus sativus*
M42	Crocetin dimethyl ester	30.53	0.32	1.07	4	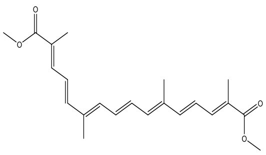	*Crocus sativus*

Table S2 Target information of related active compounds.

UniProt-ID	Target name	Gene Name	Degree
Q96IY4	Carboxypeptidase B2	CPB2	17
P11473	Vitamin D3 receptor	VDR	16
P03372	Estrogen receptor	ESR1	13
P05067	Amyloid beta A4 protein	APP	13
Q16853	Membrane primary amine oxidase	AOC3	13
P35354	Prostaglandin G/H synthase 2	PTGS2	12
P19801	Amiloride-sensitive amine oxidase	AOC1	11
P00734	Prothrombin	F2	9
P10275	Androgen receptor	AR	8
P37231	Peroxisome proliferator-activated receptor gamma	PPARG	8
O15528	25-hydroxyvitamin D-1 alpha hydroxylase, mitochondrial	CYP27B1	8
P80365	Corticosteroid 11-beta-dehydrogenase isozyme 2	HSD11B2	8
P23219	Prostaglandin G/H synthase 1	PTGS1	7
P04150	Glucocorticoid receptor	NR3C1	7
P27338	Amine oxidase [flavin-containing] B	MAOB	5
P35228	Nitric oxide synthase, inducible	NOS2	5
P05093	Steroid 17-alpha-hydroxylase/17,20 lyase	CYP17A1	5
P29474	Nitric oxide synthase, endothelial	NOS3	5
P49841	Glycogen synthase kinase-3 beta	GSK3B	4
P08235	Mineralocorticoid receptor	NR3C2	4
Q16539	Mitogen-activated protein kinase 14	MAPK14	3
Q16445	Gamma-aminobutyric acid receptor subunit alpha-6	GABRA6	3
P47989	Xanthine dehydrogenase/oxidase	XDH	3
Q12882	Dihydropyrimidine dehydrogenase [NADP(+)]	DPYD	3
P48736	Phosphatidylinositol 4,5-bisphosphate 3-kinase catalytic subunit gamma isoform	PIK3CG	2
P18031	Tyrosine-protein phosphatase non-receptor type 1	PTPN1	2
Q92731	Estrogen receptor beta	ESR2	2
P04798	Cytochrome P450 1A1	CYP1A1	2
Q16678	Cytochrome P450 1B1	CYP1B1	2
P20701	Integrin alpha-L	ITGAL	2
P28074	Proteasome subunit beta type-5	PSMB5	2
O14939	Phospholipase D2	PLD2	2
P42330	Aldo-keto reductase family 1 member C3	AKR1C3	2
P00325	Alcohol dehydrogenase 1B	ADH1B	2
Q07973	1,25-dihydroxyvitamin D(3) 24-hydroxylase,	CYP24A1	1

	mitochondrial		
Q12791	Calcium-activated potassium channel subunit alpha-1	KCNMA1	1
P07451	Carbonic anhydrase 3	CA3	1
P10253	Lysosomal alpha-glucosidase	GAA	1
P33527	Multidrug resistance-associated protein 1	ABCC1	1
P09917	Arachidonate 5-lipoxygenase	ALOX5	1
P35869	Aryl hydrocarbon receptor	AHR	1
P11712	Cytochrome P450 2C9	CYP2C9	1
P06881	Calcitonin gene-related peptide 1	CALCA	1
P00813	Adenosine deaminase	ADA	1
Q02763	Angiopoietin-1 receptor	TEK	1
P29274	Adenosine receptor A2a	ADORA2A	1
O60603	Toll-like receptor 2	TLR2	1
P00742	Coagulation factor X	F10	1
Q14790	Caspase-8	CASP8	1
P10635	Cytochrome P450 2D6	CYP2D6	1
P09960	Leukotriene A-4 hydrolase	LTA4H	1
Q16739	Ceramide glucosyltransferase	UGCG	1
P07550	Beta-2 adrenergic receptor	ADRB2	1
P35368	Alpha-1B adrenergic receptor	ADRA1B	1
P08172	Muscarinic acetylcholine receptor M2	CHRM2	1
P35348	Alpha-1A adrenergic receptor	ADRA1A	1
P23975	Sodium-dependent noradrenaline transporter	SLC6A2	1
P28223	5-hydroxytryptamine receptor 2A	HTR2A	1
Q14432	cGMP-inhibited 3',5'-cyclic phosphodiesterase A	PDE3A	1
P08913	Alpha-2A adrenergic receptor	ADRA2A	1
P08588	Beta-1 adrenergic receptor	ADRB1	1
P11229	Muscarinic acetylcholine receptor M1	CHRM1	1
P53634	Dipeptidyl peptidase 1	CTSC	1
P08183	Multidrug resistance protein 1	ABCB1	1
Q92934	Bcl2-associated agonist of cell death	BAD	1
P05771	Protein kinase C beta type	PRKCB	1

Table S3 The relationships between active compounds and potential targets.

NO.	Compound	Target
M01	Castorin	PRKCB、BAD、HSD11B2
M02	Cholesterol	ABCB1、HSD11B2、VDR、CYP27B1、NR3C1、CYP17A1、PPARG、NR3C2、ESR1、AR
M03	Salicin	CPB2、CTSC、NOS2、PTGS1、ESR1、PTGS2
M04	Salicin-qt	CPB2、DPYD、AOC1、AOC3
M05	Benzoic acid	CPB2、AOC1、DPYD、AOC3、ADH1B、MAOB
M06	Ethylphenol	CPB2、AOC1、MAOB、GSK3B、AOC3
M07	Muscopyridine	AOC3、CPB2、VDR、CHRM1、ADRB1、ADRA2A、PDE3A、HTR2A、SLC6A2、ADRA1A、CHRM2、ADRA1B、ADRB2
M08	Muscone	VDR、AOC3、CPB2、F2、PTGS2
M09	Normuscone	VDR、AOC3、CPB2、AOC1
M10	Muscopyran	UGCG、LTA4H、CYP2D6、CASP8、HSD11B2、NR3C2、F2、F10
M11	5a-Androstanedione	HSD11B2、VDR、NR3C1、CYP17A1、ESR1、AR、AKR1C3
M12	Cholest-4-ene-3-one	HSD11B2、VDR、NR3C1、CYP17A1、CYP27B1、PPARG、ESR1、AR
M13	Dehydroepiandrosterone	HSD11B2、VDR、NR3C1、CYP17A1、CYP27B1、NR3C2、ESR1、AR、AKR1C3
M14	Cholesteryl ester	HSD11B2、TLR2、NR3C1、CYP27B1
M15	Crocetin dialdehyde	AOC3、CPB2、APP、F2、PTGS2、NOS3
M16	Adenosine	ADORA2A、TEK、NOS2、F2、ESR1、PTGS2、NOS3、ADA
M17	Adenosine-qt	XDH、AOC3、AOC1、DPYD、GSK3B
M18	Anthocyanins	PTGS1、ESR1、PTGS2、MAOB
M19	Carotene-a	VDR、CYP27B1、APP
M20	Carotene	CYP27B1、APP、VDR
M21	Crocetin	PTGS1、CPB2、APP、NOS2、F2、ESR1、PPARG、PTGS2、NOS3
M22	Crocin	VDR、PLD2、CALCA、APP、PSMB5、ITGAL
M23	Crocin-qt	PTGS1、CPB2、APP
M24	Crocin1	PLD2
M25	Crocin1-qt	CPB2、F2、ESR1、PPARG、PTGS2、NOS3
M26	Crocin2	VDR、PSMB5、ITGAL
M27	Crocin2-qt	PTGS1、CPB2、APP、F2、PPARG、PTGS2、NOS3
M28	Foron	AOC3、AOC1、CPB2
M29	Isophorone	AOC3、AOC1、CPB2、GABRA6
M30	Isorhamnetin	CYP1B1、CYP1A1、MAPK14、XDH、MAOB、ESR2 NOS2、PTGS1、ESR1、AR、PPARG、PTGS2、PTPN1、GSK3B、PIK3CG
M31	Kaempferol	CYP2C9、AR、CYP1B1、CYP1A1、AHR、ALOX5、

		MAPK14、XDH、ABCC1、MAOB、ESR2、NOS2、PTGS1、ESR1、PPARG、PTGS2、PTPN1、GSK3B、PIK3CG
M32	Lycopene	VDR、APP
M33	Phytofluene	VDR、APP
M34	Picrocrocin	GAA、F2、ESR1、AR、PTGS2
M35	Picrocrocin-qt	AOC3、AOC1、GABRA6
M36	Safranal	AOC3、AOC1
M37	Stigmasterol	HSD11B2、CYP27B1、VDR、NR3C1、CYP17A1、PPARG、APP、ESR1、AR、NR3C2
M38	Catechol	CA3、AOC1、CPB2、KCNMA1、ADH1B、MAPK14
M39	β-Phorone	AOC3、AOC1、CPB2、GABRA6
M40	Tetrahydrolycopene	VDR、APP
M41	Zeaxanthin	VDR、CYP27B1、CYP24A1、APP、NR3C1
M42	Crocetin dimethyl ester	APP、CPB2、F2、PTGS2

Table S 4 Chemical information of 113 compounds and their network parameters.

Molecule-ID	Herb-name	Molecule-name	OB	CACO2	DL
1	Beaver Castoreum	2,3,5-Trimethylpyrazine	37.33	0.75	0.05
2	Beaver Castoreum	2－hydroxybenzaldehyde	32.10	0.84	0.02
3	Beaver Castoreum	4-Ethylguaiacol	60.39	1.43	0.03
4	Beaver Castoreum	4-Methylguaiacol	39.66	1.40	0.03
5	Beaver Castoreum	4-Propylguaiacol	57.31	1.36	0.04
6	Beaver Castoreum	benzyl alcohol	45.52	1.07	0.01
7	Beaver Castoreum	Borneol	83.54	1.21	0.05
8	Beaver Castoreum	castoramine	23.85	0.90	0.14
9	Beaver Castoreum	D(-)-Salicin	8.38	-1.05	0.16
10	Beaver Castoreum	D(-)-Salicin-qt	60.32	0.89	0.02
11	Beaver Castoreum	Castorin	10.87	-0.02	0.40
12	Beaver Castoreum	Salicin	8.38	-1.05	0.16
13	Beaver Castoreum	Salicin-qt	60.32	0.89	0.02
14	Beaver Castoreum	Benzoic acid	35.20	0.85	0.02
15	Beaver Castoreum	Ethylphenol	72.56	1.60	0.02
16	Beaver Castoreum	2,3,5,6-Tetramethylpyrazine	29.22	1.20	0.03
17	Moschus	Glyceryl tri-palmitate	16.29	0.72	0.19
18	Moschus	Histidine	52.17	-0.35	0.03
19	Moschus	Lysine	53.24	-0.70	0.02
20	Moschus	Phenylalanine	47.22	0.20	0.04
21	Moschus	Isoleucine	45.00	0.12	0.02
22	Moschus	Cystine	74.85	-1.34	0.05
23	Moschus	Aspartic acid	43.36	-1.14	0.02
24	Moschus	Alanine	86.91	-0.28	0.01
25	Moschus	Glycine	53.63	-0.52	0.00
26	Moschus	Proline	44.82	0.34	0.01
27	Moschus	Arginine	68.12	-0.49	0.03
28	Moschus	Estradiol	35.74	0.96	0.32
29	Moschus	Methyl palmitate	18.09	1.38	0.12
30	Moschus	Octadecanone	14.81	1.53	0.11
31	Moschus	oleodipalmitin	21.08	0.86	0.17
32	Moschus	Pentadecanoic acid ethyl ester	19.74	1.37	0.11
33	Moschus	Serine	98.47	-1.12	0.01
34	Moschus	(4S)-4-hydroxy-3,5,5-trimethylcyclohex-2-en-1-one	36.27	-0.05	0.08
35	Moschus	Cyclopentadecanone	39.54	1.39	0.09
36	Moschus	muscopyridine	15.31	1.76	0.12
37	Moschus	Muscone	36.63	1.43	0.10
38	Moschus	Normuscone	39.54	1.39	0.09
39	Moschus	Methyl,(benzoyloxy)-(9CI)	30.20	1.24	0.07

40	Moschus	Oleic acid	33.13	1.08	0.14
41	Moschus	Testosterone	14.47	0.68	0.35
42	Moschus	undec-2-ene	22.57	1.84	0.02
43	Moschus	Mus-copyran	63.08	-1.71	0.64
44	Moschus	3a-Hydroxy-5b-androstan-17-one	14.63	0.58	0.35
45	Moschus	5a-Androstanedione	14.72	0.71	0.35
46	Moschus	5α-androstane-3β,17α-diol	24.76	0.51	0.34
47	Moschus	5β-Androstan-3α,17α-diol	23.44	0.11	0.38
48	Moschus	5β-Androstan-3α,17β-diol	27.04	0.50	0.34
49	Moschus	Allantoin	106.68	-0.81	0.03
50	Moschus	cellulose	20.71	-1.96	0.03
51	Moschus	Cholest- 4 -ene- 3 - one	7.45	1.45	0.68
52	Moschus	Cyclododecanone	45.13	1.37	0.04
53	Moschus	Cyclotetradecanone	40.65	1.41	0.07
54	Moschus	Dehydroepiandrosterone	33.86	0.67	0.35
55	Moschus	Cholesteryl ester	45.15	1.59	0.39
56	Moschus	Hydroxymuscopyridine A	34.14	1.03	0.14
57	Moschus	Hydroxymuscopyridine B	13.41	1.14	0.14
58	Moschus	GLYCERYL TRIPALMATE	16.29	0.72	0.19
59	Moschus	Dipalmitolein	34.58	0.55	0.45
60	Crocus sativus	2,6,6-Trimethylcyclohex-2-ene-1,4-dione	69.41	0.65	0.04
61	Crocus sativus	3-hydroxy-beta-ionone	19.08	0.59	0.07
62	Crocus sativus	4-Hydroxy-2,6,6-trimethyl cyclohex-1-enecarbaldehyde	39.91	0.52	0.04
63	Crocus sativus	5-methyluracil	74.37	0.22	0.02
64	Crocus sativus	Adenosine	19.90	-1.52	0.18
65	Crocus sativus	Adenosine-qt	59.70	-0.26	0.03
66	Crocus sativus	anthocyanins	55.28	1.80	0.11
67	Crocus sativus	campesterol	9.44	1.33	0.71
68	Crocus sativus	Carotene-a	36.04	2.30	0.58
69	Crocus sativus	Carotene	37.71	2.27	0.59
70	Crocus sativus	crocetin	44.03	0.56	0.26
71	Crocus sativus	crocin	2.48	-4.40	0.12
72	Crocus sativus	crocin-qt	33.44	0.44	0.26
73	Crocus sativus	crocin1	3.01	-4.89	0.12
74	Crocus sativus	crocin1-qt	45.07	0.46	0.26
75	Crocus sativus	crocin2	2.90	-3.18	0.21
76	Crocus sativus	crocin2-qt	41.40	0.46	0.26
77	Crocus sativus	crocin3	5.98	-2.30	0.44

78	Crocus sativus	crocin3-qt	33.44	0.43	0.26
79	Crocus sativus	crocin4	14.86	-0.71	0.61
80	Crocus sativus	crocin4-qt	29.38	0.74	0.29
81	Crocus sativus	Crocusatin G	45.25	0.63	0.06
82	Crocus sativus	Crocusatin H	29.86	-0.17	0.08
83	Crocus sativus	Crocusatin I	67.89	0.30	0.05
84	Crocus sativus	Crocusatin B	53.68	-0.05	0.05
85	Crocus sativus	Crocusatinsf	48.94	0.49	0.08
86	Crocus sativus	Crocusatinc	45.73	0.49	0.04
87	Crocus sativus	Foron	32.29	1.27	0.02
88	Crocus sativus	isophorone	44.98	1.28	0.03
89	Crocus sativus	isorhamnetin	7.02	0.12	0.31
90	Crocus sativus	Kaempferol 3,7,4'-triglucoside	3.08	-3.87	0.34
91	Crocus sativus	Kaempferol 3,7,4'-triglucoside-qt	62.20	0.21	0.24
92	Crocus sativus	Kaempferol 3-sophoroside-7-glucoside	3.25	-3.33	0.35
93	Crocus sativus	Kaempferol 3-sophoroside-7-glucoside-qt	62.21	0.22	0.24
94	Crocus sativus	kaempferol	69.61	0.15	0.24
95	Crocus sativus	Kaempferol-3-sophoroside	4.51	-2.57	0.71
96	Crocus sativus	Kaempferol-3-sophoroside-qt	62.82	0.23	0.24
97	Crocus sativus	Linoleic acid	41.90	1.15	0.14
98	Crocus sativus	lycopene	36.18	2.41	0.51
99	Crocus sativus	Methylparaben	53.37	0.77	0.03
100	Crocus sativus	Nonadecanol	12.36	1.36	0.14
101	Crocus sativus	phytofluene	43.18	2.28	0.50
102	Crocus sativus	picrocrocin	22.42	-0.82	0.23
103	Crocus sativus	picrocrocin-qt	38.42	0.71	0.04
104	Crocus sativus	Safranal	39.81	1.42	0.04
105	Crocus sativus	stigmasterol	43.83	1.31	0.76
106	Crocus sativus	catechol	29.35	1.08	0.02
107	Crocus sativus	3,5-Dimethylbenzaldehyde	27.74	1.31	0.04
108	Crocus sativus	beta-Phorone	46.35	1.30	0.03
109	Crocus sativus	tetrahydrolycopene	33.02	2.41	0.49
110	Crocus sativus	Zeaxanthin	14.18	1.04	0.54
111	Crocus sativus	Crocetin dimethyl ester	30.53	1.07	0.32
112	Crocus sativus	Crocetin dialdehyde	38.46	1.16	0.19
113	Beaver Castoreum/Moschus	cholesterol	37.87	1.31	0.68
